# Human Brain Expansion during Evolution Is Independent of Fire Control and Cooking

**DOI:** 10.3389/fnins.2016.00167

**Published:** 2016-04-25

**Authors:** Alianda M. Cornélio, Ruben E. de Bittencourt-Navarrete, Ricardo de Bittencourt Brum, Claudio M. Queiroz, Marcos R. Costa

**Affiliations:** ^1^Brain Institute, Federal University of Rio Grande do NorteNatal, Brazil; ^2^Department of Morphology, Center of Biosciences, Federal University of Rio Grande do NorteNatal, Brazil; ^3^Department of Physiology, Institute of Biological Sciences, Federal University of Juiz de ForaJuiz de Fora, Brazil

**Keywords:** human evolution, brain size, fire control, thermal processing of food, cooking

## Abstract

What makes humans unique? This question has fascinated scientists and philosophers for centuries and it is still a matter of intense debate. Nowadays, human brain expansion during evolution has been acknowledged to explain our empowered cognitive capabilities. The drivers for such accelerated expansion remain, however, largely unknown. In this sense, studies have suggested that the cooking of food could be a pre-requisite for the expansion of brain size in early hominins. However, this appealing hypothesis is only supported by a mathematical model suggesting that the increasing number of neurons in the brain would constrain body size among primates due to a limited amount of calories obtained from diets. Here, we show, by using a similar mathematical model, that a tradeoff between body mass and the number of brain neurons imposed by dietary constraints during hominin evolution is unlikely. Instead, the predictable number of neurons in the hominin brain varies much more in function of foraging efficiency than body mass. We also review archeological data to show that the expansion of the brain volume in the hominin lineage is described by a linear function independent of evidence of fire control, and therefore, thermal processing of food does not account for this phenomenon. Finally, we report experiments in mice showing that thermal processing of meat does not increase its caloric availability in mice. Altogether, our data indicate that cooking is neither sufficient nor necessary to explain hominin brain expansion.

## Introduction

Human evolution is marked by a significant increase in the total brain size relative to body size, referred to as encephalization. Although increased encephalization is a clear hallmark of human cognitive and cultural evolution, there is little consensus on the causes of such phenomenon. In part, this lack of agreement reflects the difficulty to test directly numerous hypotheses proposed to explain the brain growth in the hominin lineage.

Studies on human brain evolution have been largely based upon two main lines of evidence: (i) fossil records, termed paleoneurology; and (ii) indirect evidence coming from anatomical, physiological, and behavioral comparison between humans and closely related extant primates, such as chimpanzee. While the former allows inferences about the total brain volume of extinct hominins, comparisons between existing primates permit a more detailed analysis on how gross and microscopic organization of the brain correlates with different behaviors, thus allowing some inferences about anatomic and functional aspects of the brain. However, it is important to bear in mind that extant living species, such as chimpanzee, gorilla, and macaque, are the endpoints of their own evolutionary lines and not our ancestors (Holloway, 1968). Therefore, the combination of direct and indirect evidence is mandatory to develop a better understanding of when and how the human brain evolved.

Based on such “direct” and “indirect” evidence, many different theories have been proposed to explain the disproportionate growth of the brain in the human lineage, considering the high energetic cost of larger brains (Mink et al., 1981). The expensive-tissue hypothesis, for example, explains brain evolution by proposing a tradeoff between the size of the brain and that of the digestive tract, which is smaller than expected for a primate of our body size (Aiello and Wheeler, 1995). However, an important study, in which body mass was controlled for fat-free body mass, failed to find negative correlations between the relative size of the brain and the digestive tract (or any other expensive organ such as heart, lungs, kidneys, spleen, or liver) for the 100 species of mammals analyzed, including 23 primates (Navarrete et al., 2011).

Recently, it has been proposed that energetic constraints could impose a tradeoff between body and brain growth during primates' evolution (Fonseca-Azevedo and Herculano-Houzel, 2012). According to this hypothesis, limitations for calories obtained during foraging hours would impose a selective pressure on body vs. brain growth. In that case, primates with larger body size would present proportionally smaller brain mass due to dietetic restraints. Human ancestors, but not other primates, are supposed to have bypassed such constraints by cooking. This would increase the available calorie content in the food (Carmody et al., 2011; Fonseca-Azevedo and Herculano-Houzel, 2012).

In this work, we revisit the correlations between brain size and cooking by means of archeological and neuroanatomical data, as well as new metabolic data on the energetic content of raw and cooked meat. We show that large primate encephalization was reached millions of years before the widespread control of fire, a pre-requisite for cooking, and provide evidence indicating that early hominins were likely to obtain enough calories from raw meat to afford for the size of their brains.

## Materials and methods

### Mathematical model

Kleiber's law (Kleiber, 1947) was used to determine body's daily energetic need (*E*_*BD*_) according to the equation:

(1) $$\begin{array}{l} E_{BD}=Z\times \;{M_{BD}}^{0.75} \end{array}$$

where *Z* is a correction factor that varies among species and according to physical activity and *M*_*BD*_ is the body mass. To maintain proper physiological functions and stable body mass during no exercise or sedentary conditions, we have used Harris and Benedict (1918) equation, in which:

(2) $$\begin{array}{l} E_{BD}=BMR\times 1.2 \end{array}$$

where *BMR* is the basal metabolic rate of the body. According to this approach (Harris and Benedict, 1918), *BMR* can be calculated by using the following equation (for men):

(3) $$\begin{array}{l} BMR=66+\left(13.7\times w\right)+\left(5\times h\right)-\left(6.8\times a\right) \end{array}$$

where *w* is weight (in kilogram), *h* is height (in centimeter), and *a* is age (in years). Combining Equations (1) and (2), one obtains:

(4) $$\begin{array}{l} BMR\times 1.2=Z\times {M_{BD}}^{0.75} \end{array}$$

Using Equations (3, 4), it is possible to calculate the *Z* factor for an early male hominin (70 kg, 1.6 m and 30 years-old) under resting conditions (Harris and Benedict, 1918; Kleiber, 1947):

$$\begin{array}{c} BMR\times 1.2=Z\times {M_{BD}}^{0.75}\\ \left[66+\left(13.7\times 70\right)+\left(5\times 160\right)-\left(6.8\times 30\right)\right]\times 1.2=Z\times 70^{0.75}\\ 1621\times 1.2=Z\times 24.2\\ Z=80.3 \end{array}$$

Previous work (Fonseca-Azevedo and Herculano-Houzel, 2012) has adopted *Z* = 70, likely underestimating daily energetic need (see Discussion). Here, we expect that early hominins would have at least moderate physical activity (e.g., at least 60 min/day, 5 times per week) and, therefore, would have an energetic need 1.55 times the *BMR* (Harris and Benedict, 1918). Updating Equation (4) to:

(5) $$\begin{array}{l} BMR\times 1.55=Z\times {M_{BD}}^{0.75} \end{array}$$

leads to

$$\begin{array}{c} \left[66+\left(13.7\times 70\right)+\left(5\times 160\right)-\left(6.8\times 30\right)\right]\times 1.55=Z\times 70^{0.75}\\ 1621\times 1.55=Z\times 24.2\\ Z=103 \end{array}$$

Unless otherwise stated, this *Z* factor value was used in all the following calculations.

Brain volumes for different species were obtained from previous publications (see Table 1). To enable comparisons between the present and previous studies, the estimated number of neurons per brain was the same used by Fonseca-Azevedo and Herculano-Houzel (2012). Graphics and statistical tests were performed using Matlab program (version 7, R14, Mathworks) or GraphPad Prism version 5.00 (San Diego California USA, www.graphpad.com). The confidence interval was set to 95%.

**Table 1:** **Brain volumes and body mass of hominid species throughout evolution**.

**Taxon**	**Fossile evidence**	**Time (MYA)**	**Brain volume (cc)**	**Body mass (Kg)**	**References**
G. gorilla	(Afar, Ethiopia, 2007)	10,0–12,0	420–680	150	Suwa et al., 2007
Pan troglodytes	(East of Great Rift Valley, Kenya, 2005)	4,0–5,0	320–480	50	McBrearty and Jablonski, 2005
Ardipithecus ramidus	(Asduma, Ethiopia, 1994)	4,6–4,3	350	27	Semaw et al., 2005
Australopithecus anamensis	(Kanapoi, Kenya, 1994)	4,2–3,9		50	Leakey et al., 1995
Aus. afarensis (Lucy)	(Afar, Ethiopia, 1974)	3,7–3	375–550	37	Johanson and White, 1980
Aus. africanus	(Taung, South Africa, 1924)	3–2,4	460	35	McHenry, 1992
Aus. Garhi	(Awash, Ethiopia, 1996)	2–3,0	450	unknown	Asfaw et al., 1999
Aus. sediba	(Malapa, South Africa, 2008)	1,9–1,8	420–450	unknown	Berger et al., 2010
Paranthropus aethiopicus	(Omo River, Ethiopia, 1968)	2,6–2,2	400–490	37	Falk et al., 2005
Par. Boisei	(Olduvai, Tanzania, 1959)	2,3–1,2	480–515	50	Leakey et al., 1961, 1964
Par. robustus	(Kromdraai, South Africa, 1938)	2–1,3	400–450	36	Curnoe et al., 2001
Homo rudolfensis	(Koobi Fora, Kenya, 1972)	2,4–1,8	520–750	45	Leakey and Wood, 1973
H. habilis	(Olduvai, Tanzania, 1962)	2,4–1,4	510–650	35	Leakey, 1966; McHenry, 1992
H. ergaster	(Koobi Fora, Kenya,1975)	1,9–1	800–880	60	Swisher et al., 1994
H. georgicus	(Dmanisi, Georgia, 2002)	1,8	680–770	55	Vekua et al., 2002
H. erectus	(Yuanmoun, China, 1965)	1,8–0,3	940–1200	60	Pu et al., 1977; Leigh, 1992
H. erectus erectus	(Trinil, Java, Indonesia, 1892)	1,8–0,3	850–1200	unknown	Dubois, 1894
H. lantianensis	(Lantian, Shaanxi, China, 1964)	1–0,53	780–1120	unknown	Woo, 1964, 1965
H. pekinensis	(Zhoukoudian, Peking, China, 1927)	0,5–0,25	1075	unknown	Shen et al., 2009
H. antecessor	(Atapuerca, Burgos, Spain, 1994)	0,95–0,75	1100–1150	75	Bermudez de Castro et al., 1997
H. cepranensis	(Ceprano, Lazio, Italy, 1994)	0,7–0,43	1200	unknown	Manzi et al., 2001
H. rhodesiensis	(Broken Hill, now Kabwe, Zambia, 1921)	0,63–0,16	1250–1320	unknown	Murrill, 1975; Rightmire, 1983; Conroy et al., 2000
H. heidelbergensis	(Heidelberg, Germany, 1908)	0,65–0,2	1100–1370	80–100	Schoetensack, 1908; Arsuaga et al., 1993
H. neanderthalensis	(Dusseldorf, Germany, 1856)	0,45–0,028	1200–1500	70–90	King, 1864; Holloway, 1997; Helmuth, 1998
H. sapiens	(Omo River, Ethiopia, 1967)	0,195	1250–1400	70	Holloway, 1997; White et al., 2003; McDougall et al., 2005

### Archeological data

We classified the evidence of fire control (by hominins) in strong (SE), weak (WE), very weak (VWE), and non-existent (NE) according to James (1989). Briefly, we classified archeological findings into two main categories: (1) Suggestive of human action: fire-hardened wood (FHW), burned bones (BB), burned shells (BS), burned food (BF), fire-cracked rock (FR), burned lithics (BL), and hearth (H); and (2) Potentially accidental: charcoal (C), burned deposit (BD), baked clay (BC), ashes (A), reddened area (RA). Next, we examined the association of these findings with human bones (HB) and tools (T). Evidence of human control of fire was classified as strong when at least one archeological finding of fire was associated with human bones or tools; weak evidence was classified when the association was not clear; and very weak evidence for sites where only potentially accidental findings of fire were present, with no association with human bones or tools (Table 2).

**Table 2:** **Archeological evidences of fire control by hominids**.

**Archeological site**	**Date (MYA)**	**Country**	**Kind of evidence**					**Association**	**Classification**	**References**
Yuanmou	1,7	China	C	BB?				None	VWE	James, 1989
Koobi Fora	1,55	Kenya	BL?					None	VWE	James, 1989
Koobi Fora	1,4	Kenya	RA	BL?				None	VWE	James, 1989
Chesowanja	1,4	Kenya	BC					None	VWE	James, 1989
Swartkrans	1,2	South Africa		BB				not clear	WE	Brain and Sillen, 1988
Wonderwerk Cave	1	South Africa	A	BB	T			not clear	WE	Berna et al., 2012
Gesher Benot Yaakov	0,79	Israel	H	T				Clear	SE	Alperson-Afil et al., 2009
Zhoukoudian	0,5	China	BD	BL				None	VWE	Weiner et al., 1998
Atapuerca	0,6	Spain	H	T	HB			Clear	SE	Arsuaga et al., 1993
Zhoukoudian	0,45	China	H	BB	BF			not clear	WE	Wu, 1999
Schöningen	0,4	Germany	A	C	FHW	BF		clear	SE	Thieme, 1997
Qesem Cave	0,38	Israel	H	BB	BF	HB	T	clear	SE	Karkanas et al., 2007
Bajondillo	0,15	Spain	H	BF	BB	HB	T	clear	SE	Cortés-Sanchez et al., 2011
Bolomar	0,13	Spain	H	BF	T	HB		clear	SE	Blasco, 2008

SE, strong evidence; WE, weak evidence; VWE, very weak evidence; NE, non-existent evidence; FHW, fire-hardened wood; BB, burned bones; BS, burned shells; BF, burned food; FR, fire-cracked rock; BL, burned lithics; H, hearth; C, charcoal; BD, burned deposit; BC, baked clay; A, ashes; RA, reddened area; HB, human bones; T, tools.

### Energetic gain from raw and cooked meat

To directly test whether thermal processing of meat could increase the energetic gain we used the protocol described by Carmody et al. (2011). Briefly, adult male Swiss mice weighing 31–51 g, were fed with raw or cooked beef eye round (B. Taurus). Meat was acquired from a local supermarket (Nordestão, Natal, RN) and kept in a refrigerator at 4°C during all tests days. Meat was cut in cubes (~1.5 cm per side) and weighted in portions (30.0 ± 0.5 g). Cooked meat samples were placed into Petri dishes and roasted in preheated convection oven at 220°C for 15 min. Raw meat samples were kept in the refrigerator for the same time of roasting. Diets were kept at room temperature to equilibrate before feeding started (Supplementary Figure 1).

Mice housed individually received water and the experimental diet provided in Petri dishes, during all experiment period. Cages, liners, diets, and water were changed daily. Mice were fed for four consecutive days with either raw (RW) or cooked (CK) meat diets. Both diets were offered at the same time each day to all animals. Body weight was recorded daily. Residual food from the previous 24 h was collected and weighed for analysis. All experiments were carried out in accordance with Brazilian and international laws for animal care and were approved by the Institutional Animal Care and Use Committee (IACUC).

## Results

### Foraging efficiency is important to sustain large number of neurons

Recent work suggests that the energetic cost of the brain is proportional to its number of neurons. Hence, increasing the number of neurons during primate evolution would impose a metabolic constraint to the body and brain sizes (Fonseca-Azevedo and Herculano-Houzel, 2012). Mathematical models assuming that the caloric intake per hour among hominins scales as a function of the body mass (*M*_*BD*_) with an exponent equals to 0.526 (*M*_*BD*_^0.526^) support this conclusion (Fonseca-Azevedo and Herculano-Houzel, 2012). However, this function (*M*_*BD*_^0.526^) derives from estimates comprising the daily hours of foraging and body masses of 10 non-human primates with a largely vegetarian- or frugivorous-based diet (Richards, 2002). Worth noting, this model does not take into consideration any other probable changes in the diet of early hominins, such as increased consumption of animal protein and fat. Therefore, we set out to re-evaluate whether metabolic limitations would impose a tradeoff between body size and the number of brain neurons in human evolution, assuming that foraging efficiency does not vary according to the body mass.

Firstly, we assumed that the daily energy intake (*E*_*IN*_) is directly proportional to the number of foraging hours (*H*) and the average amount of calories obtained per hour of foraging, which represents the foraging efficiency (*Q*). Next, we calculated the energetic need of the body (*E*_*BD*_) with moderate activity (spent during foraging) per species using the Kleiber's law (*E*_*BD*_ = 103 × *M*_*BD*_^0.75^, see Equation 1, Materials and Methods). In the steady state, *E*_*IN*_ = *E*_*BD*_ or *H* × *Q* = 103 × *M*_*BD*_^0.75^. To separate the energetic costs of the brain and the body, we subtracted the brain mass (*M*_*BR*_) from *M*_*BD*_ in Equation (1) and included the energy of the brain (*E*_*BR*_) as a new variable:

(6) $$\begin{array}{l} H\times Q=103\times {\left(M_{BD}-M_{BR}\right)}^{0.75}+E_{BR} \end{array}$$

Since *M*_*BD*_ includes *M*_*BR*_, we have that

(7) $$\begin{array}{l} M_{BD}-M_{BR}=\left(1-r\right)\times M_{BD} \end{array}$$

where *r* = *M*_*BR*_/*M*_*BD*_, i.e., the ratio between brain and body size. Finally, assuming that the energetic cost of the brain is a linear function of its number of neurons and that the energetic cost of each neuron is 6 × 10^−9^ kcal per day in different species (Herculano-Houzel, 2011) we can derive the equation:

(8) $$\begin{array}{l} H\times Q=103\times {\left(\left(1-r\right)\times M_{BD}\right)}^{0.75}+\left(N\times 6\times 10^{-9}\right) \end{array}$$

where *N* is the total number of neurons.

**Figure 1:**
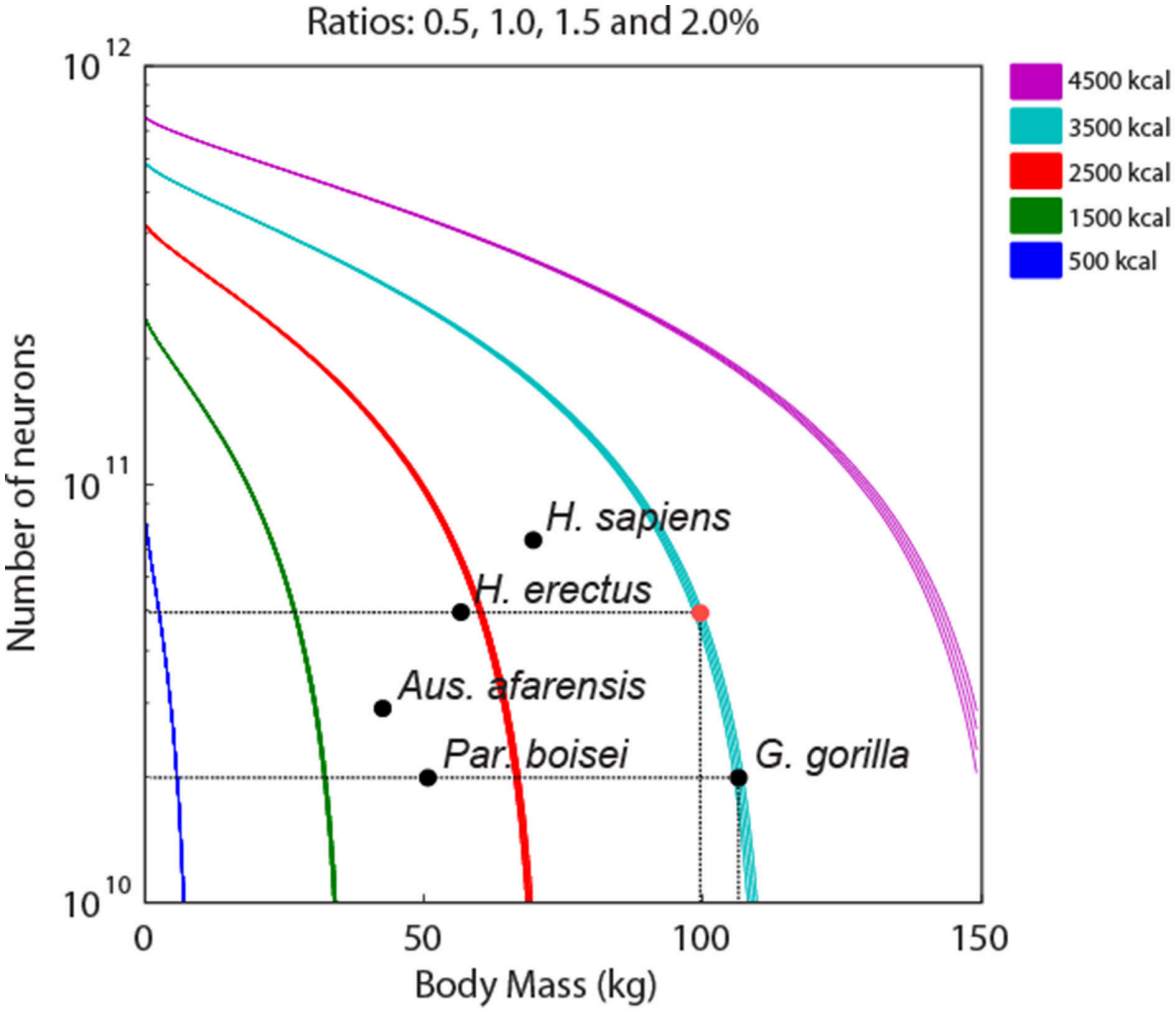
**Theoretical effects of foraging efficiency over the number of brain neurons related to body weight**. The graphic predicts the number of neurons in different foraging efficiencies, 500 (dark blue), 1500 (green), 2500 (orange), 3500 (light blue), and 4500 (purple) kcal per day. Ratios of brain and body mass vary from 0.5 to 2%. Species of primates are indicated in the graphic (black circles). Observe that small variations in the body mass are associated with dramatic increases in the number of neurons. A primate with a foraging efficiency of 3500 kcal, such as the gorilla, could easily afford the same number of neurons as a *Homo erectus* by simply reducing its weight in about 10 kg (red circle).

Using Equation (8), we first simulated how the number of neurons varies as a function of body mass for five different values of *E*_*IN*_ (500–4500 kcal/day) and four values of the ratio (*r*) *M*_*BR*_/*M*_*BD*_ (Figure 1). Interestingly, the ratio *M*_*BR*_/*M*_*BD*_ did not influence significantly the number of neurons in the brain (each *r*'s function is barely discernible from each other). In contrast, increasing the daily energy intake by 1000 kcal significantly boosted the number of neurons afforded in a body, irrespective of its mass. Thus, this simulation suggests that energy intake could be a limiting factor for an allometric scaling of body size and the number of neurons in the brain. Moreover, it also shows that species obtaining a given amount of calories per day could vary their number of brain neurons in the order of billions without significant changes in their body mass. For instance, a gorilla weighing 110 kg would need 3695 kcal/day to sustain a brain with 33 billion neurons (Figure 1). By reducing its weight to 100 kg (~10% reduction), the same gorilla could likely sustain a brain with 60 billion neurons (similar to the inferred number for *Homo erectus*) while maintaining the same amount of energy intake (red dot in the figure). These data suggest that the appealing hypothesis of a tradeoff between body mass and the number of brain neurons imposed by dietary constraints during primate evolution is unlikely.

**Figure 2:**
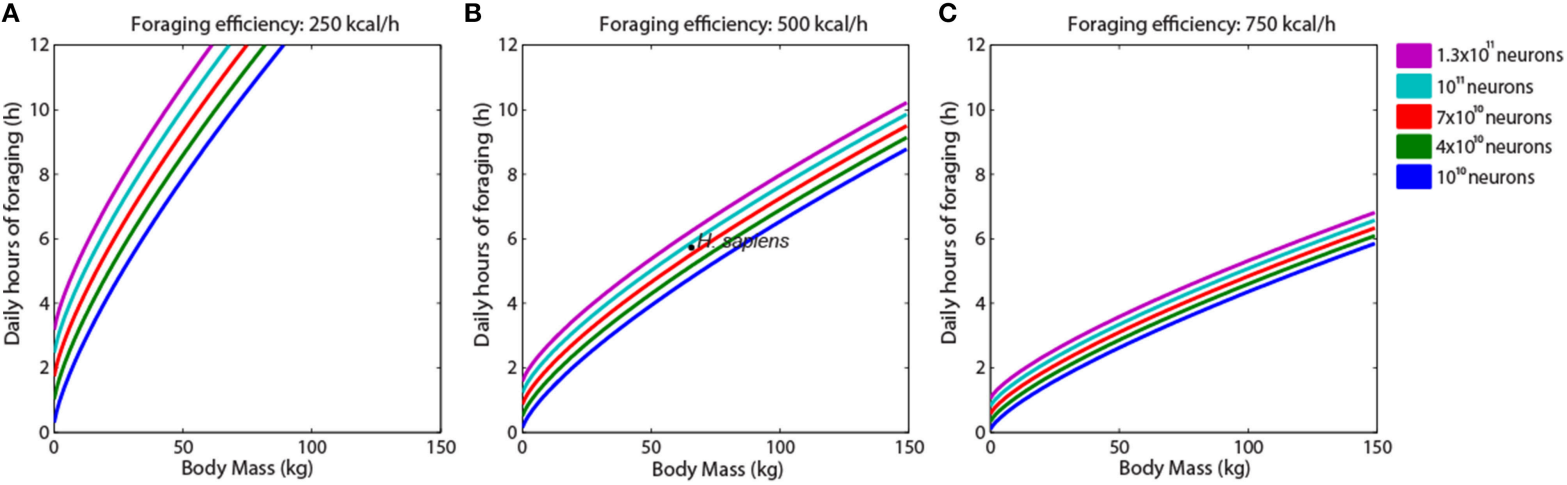
**Augmented foraging efficiency allows increases in body weight, saving daily hours of feeding, and maintaining a great number of neurons**. **(A–C)** Graphics show variations in foraging hours related to body mass for primates supporting 10 (dark blue), 40 (green), 70 (orange), 100 (light blue), or 130 (purple) billion of neurons, in three different foraging efficiencies: 250 kcal/h **(A)**, 500 kcal/h **(B)**, or 750 kcal/h **(C)**. Observe that increasing the foraging efficiency to 750 kcal/h, primates could easily weigh more than 100 kg and have 100 billion neurons spending >5 h in foraging.

Next, we simulated the number of foraging hours (*H*) as a function of body mass (*M*_*BD*_) to five values of *N* (10–130 billion of neurons) and three values of foraging efficiency (*Q*) (Figure 2). We observed that increasing foraging efficiency would have a dramatic effect on the number of foraging hours necessary to sustain a high number of neurons even in species with a great body mass. For instance, a *homo sapiens* weighing 70 kg could easily maintain 10^11^ neurons through 5 h foraging with an efficiency of 500 kcal/h (Figure 2B). Moreover, increased body weight affects foraging hours more strongly than augmented number of neurons, especially when foraging efficiency is low (Figure 2A). For high foraging efficiencies (500 and 750 kcal), subtle increases in the number of foraging hours could be sufficient to sustain increases of one order of magnitude in the number of neurons per brain, maintaining the same body weight (Figures 2B,C). Thus, the present model suggests that brain size evolution in the human lineage would heavily depend on strategies to increase foraging efficiency, rather than on a tradeoff between brain and body size. In fact, we observed no correlation between brain volumes and body masses for 16 species of primates analyzed in this work (Supplementary Figure 2B).

### Onset of human brain size expansion does not match control of fire and cooking

Which strategies early hominins may have developed to increase their foraging efficiency? Recently, it has been suggested that thermal processing of food could be the turning point in human brain evolution once it would allow increased foraging efficiency (i.e., increasing the energy content per amount of food) without a significant increase in foraging time (Carmody et al., 2011; Fonseca-Azevedo and Herculano-Houzel, 2012). Obviously, thermal processing of food required the control of fire by early hominins. However, human control of fire and its association with cooking at the onset of human brain expansion is highly controversial.

The earliest evidence of fire in archeological records dates back approximately 1 million years ago (MYA). In 1988, Brain and Sillen described the presence of burnt bones inside the Swartkrans caves (South Africa). In spite of evidence of cut-marked bones indicating butchery, those authors are cautious, stating that fire was not necessarily used for cooking (Brain and Sillen, 1988). More recently Berna and collaborators found burned bones and ashes from plants at the site of Wonderwerk cave (Northern Cape Province, South Africa). Yet, no clear association with human activity was described (Berna et al., 2012). Similarly, archeological data in the localities of Yanmou (China), Chesowanja and Koobi Fora (Kenya, Africa) 1.5 MYA do not support the human origin of the fire remains (James, 1989). Weiner et al. (1998) analyzed evidence of the use of fire in sediments accumulated from about 500,000–200,000 years ago (YA) at Zhoukoudian, China and concluded that no inference drawn from the human origin of those deposits is clear-cut. The complexity of fossil deposits commonly leads to misinterpretations. The origin of such fires might be attributed for instance to the fortuitous association between hominins action and natural causes like lightning or flames from volcanic eruptions. Furthermore, burning remains could have been carried down into caves by streams of water or seismic fractures. Weiner et al. (1998) point out the absence of ashes and charcoal and also the lack of evidence of human action in those sites.

A different scenario arises regarding the site of Gesher Benot Ya'aqov in Israel (Alperson-Afil et al., 2009). Hearths remains kept in the same area through several generations of hominins are estimated to be from 790,000 to 690,000 YA. In those sites, the presence of burned nuts is suggestive of the use of fire by humans, although it has also been associated with the use of lithic tools. Notwithstanding the clear presence of human-made fire, its goal is yet unclear. Alternative applications may include illumination, heating or even an approach to keep predators and insects away.

In favor of earlier human control over fire, it has been suggested that no occupation of Europe by hominins would be feasible without it. Nevertheless, Roebroeks and Villa (2011) show that “*surprisingly, evidence for use of fire in the Early and early Middle Pleistocene of Europe is extremely weak. Or, more exactly, it is nonexistent, until* ~*300,000–400,000 YA.*” According to these authors, the oldest evidence of fire use in Europe would be present at Beeches Pit (England) and Schöningen (Germany), around 400,000 YA. Arsuaga et al. (1993) provide older data in Sierra de Atapuerca (Spain), where *H*. *heidelbergensis* fossil records dated 600,000 YA, are associated with hearths. More recently Karkanas et al. (2007) have described consistent use of fire in the cave of Qesem (Israel), between 382,000 and 200,000 YA, for several purposes, including cooking food. Thereafter, the fire use by hominins is broadly documented throughout the Old World (James, 1989). There are also interesting cases where fire was used with aims unrelated to cooking, such as for making stones more workable, observed for the first time in the Stillbay culture (Cape Province, South Africa), 164–72,000 YA (Brown et al., 2009). Regarding the specific fire use for cooking purposes, it is reasonable to accept its archeological onset 700,000 YA at the localities of Gesher Benot Ya'aqov.

However, Foley and Gamble (2009) identified the fire control with cooking purposes as early as 800,000 YA. Wu (1999) argues that *H. erectus pekinensis* was able to cook in the cave of Getzetang (in the complex of Zhoukoudien caves) around 450,000 YA. A little later, according to Thieme (1997), *H. heidelbergensis* hunted and cooked horses in Schöningen, Northern Germany, which would represent the first collective hunts, around 400,000 YA. Meanwhile, hominins from Qesem (Israel) cooked deer and turtles 400,000 YA, a practice kept throughout 200,000 YA at the same locality (Karkanas et al., 2007).

In Spain, Cortés-Sanchez et al. (2011) describe how *H. neanderthalensis* ate cooked mussels in the cave of Bajondillo, Malaga, 150,000 YA, while Blasco (2008) reports the practice of cooking turtles in their shells, which were subsequently broken with stones, around 130,000 YA in the cave of Bolomar, Valencia.

In short, according to the archeological data available, it is reasonable to assume that fire control by hominins occurred throughout the last 1 MYA and, during the first half of such period, the evidence is sparse (Table 2). Consistent use of this element, likely associated with cooking, represents a slow process with an onset around 790,000 YA. The truly widespread use of fire control takes place with our Neanderthal “cousins,” around 450,000 YA, and our present species has finally universalized it.

**Figure 3:**
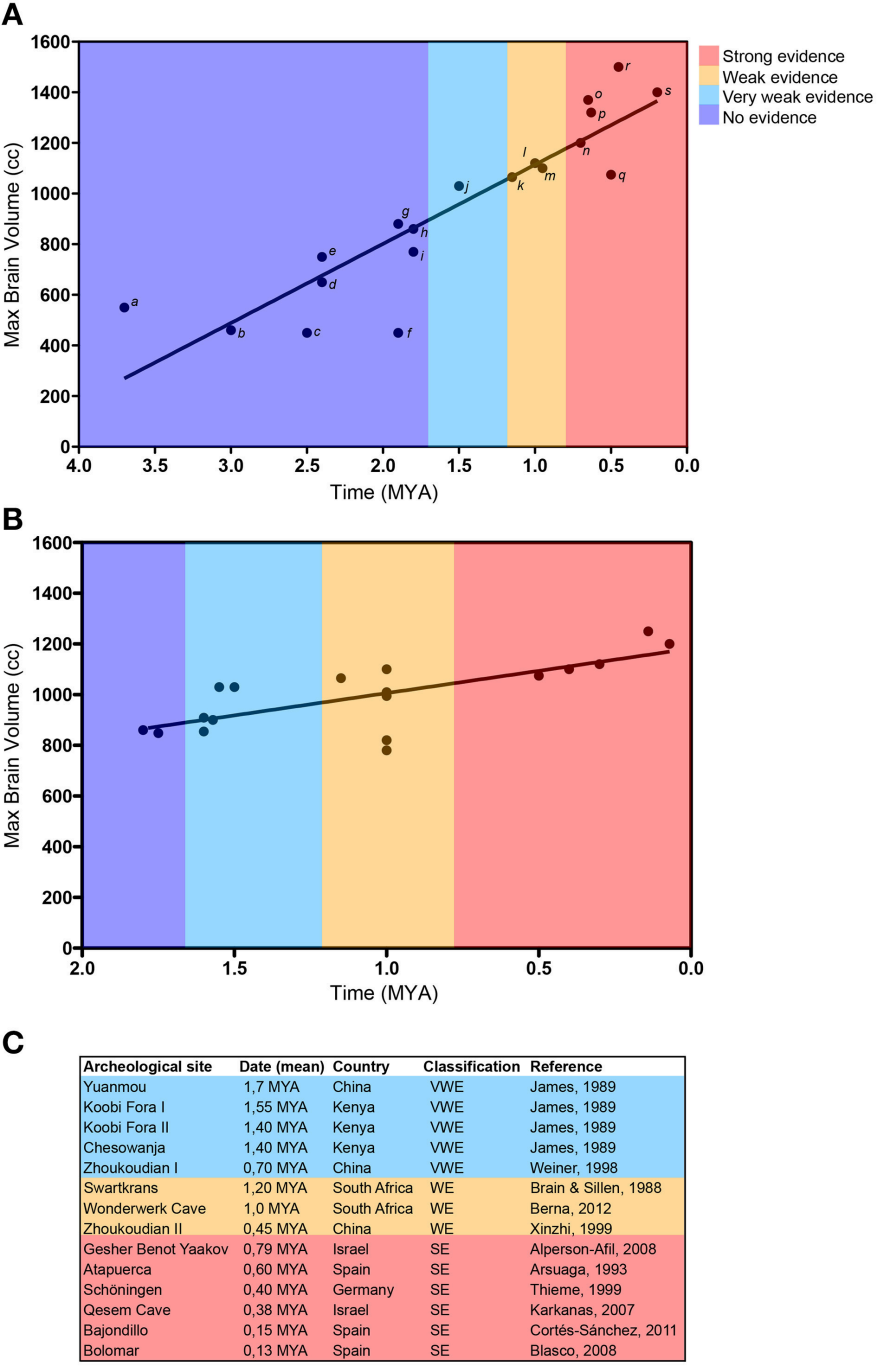
**Increase in the brain size during human evolution is independent of fire control. (A)** Maximal brain volume of different hominin species related to their oldest possible time of origin (Table 1). Data indicate that brain volume increased linearly in time (*R*^2^ = 0.8032; *p* < 0.0001). Colors represent the strength of archeological evidence supporting cooking by hominins throughout the time (**C**; see also Table 2). **(B)** Maximal brain volume of different *Homo erectus* fossils related to their oldest possible time of origin (Table 3). Observe that similar brain volumes of fossils dated from periods with weak and strong evidence for human control of fire. **(C)** Summary of archeological evidence used to classify the strength of data supporting fire control in hominin lineage. Observe that strong evidence of fire control is present only in the last 790,000 years and archeological data becomes more prominent in the last 400,000 year with *Homo neanderthalensis* and *Homo sapiens*. Also, note that species with a maximal brain volume, as large as modern humans', such as *Homo erectus*, appear at times when no evidence of fire control is present. Legends: a) *Aus. Afarensis*; b) *Aus. Africanus*; c) *Aus. garhi*; d) *Aus. sediba*; e) *Homo rudolphensis*; f) *Homo habilis*; g) *Homo ergaster*; h) *Homo georgicus*; i) *Homo erectus* (Modjokento, Indonesia); j) *Homo erectus* (Sangiran I, Indonesia); k) *Homo erectus* (Olduvai, Tanzania); l) *Homo lantianensis*; m) *Homo antecessor*; n) *Homo cepranensis*; o) *Homo rhodesiensis*; p) *Homo heidelbergensis*; q) *Homo pekinensis*; r) *Homo neanderthalensis*; s) *Homo sapiens*.

Based on these archeological evidence, we correlated maximal brain volume of different hominins species according to their time of appearance and the strength of evidence of their fire control (Figure 3). Firstly, we observed a linear brain volume increase in the human evolutionary lines during the last 4 millions of years (MY) (Figure 3A and Supplementary Figure 2A). It suggests that no evolutionary leap occurred in the human ancestry, but rather a steady and regular process took place. More importantly, adding evidence of fire control does not improve the description of brain evolution (Figure 3A). Indeed, species with large brains appeared at periods when no evidence of fire control exists (*H. ergaster*) or evidence of fire is very weak (*H. antecessor* and *H. latianensis*). Interestingly, brain volumes of distinct fossils of *H. erectus* dated from periods with weak and strong evidence for human control of fire display very similar brain volumes (Figure 3B). These data suggest that thermal processing of food is unlikely to explain the increase in foraging efficiency necessary to evolve large brains, or alternatively, brain increase results from still unknown evolutionary pathways. Therefore, archeological evidence of fire control in the human lineage does not support the view that thermal processing of food contributed to the evolution of the human brain by increasing foraging efficiency.

**Table 3:** **Maximal brain volume of ***Homo erectus*** fossils**.

**Location**	**Time (MYA)**	**Brain volume (cc)**
Modjokerto, Indonesia	1,8	860
Koobi Fora I, Kenya	1,75	848
Nariokotome, Kenya	1,6	880–909
Trinil I, Indonesia	1,6	855
Koobi Fora II, Kenya	1,57	900
KNM-ER 42700, Kenya	1,55	1030
Sangiran I, Indonesia	1,5	1030
Olduvai, Tanzania	1,15	1065
Daka, Etyopia	1	995
Buia, Eritrea	1	820
Sangiran II, Indonesia	1	1010
Lantian, Shaanxi, China	1	780
Sangiran III, Indonesia	1	1100
Zhoukoudian, China	0,5	980–1075
Trinil II, Indonesia	0,4	1100
Dali, Shaanxi, China	0,3–0,26	1120
Solo, Java, Indonesia	0,14–0,12	1015–1250
Ngandong, Indonesia	0,2–0,07	917–1200

### Raw and cooked meat bears similar energetic contents

**Figure 4:**
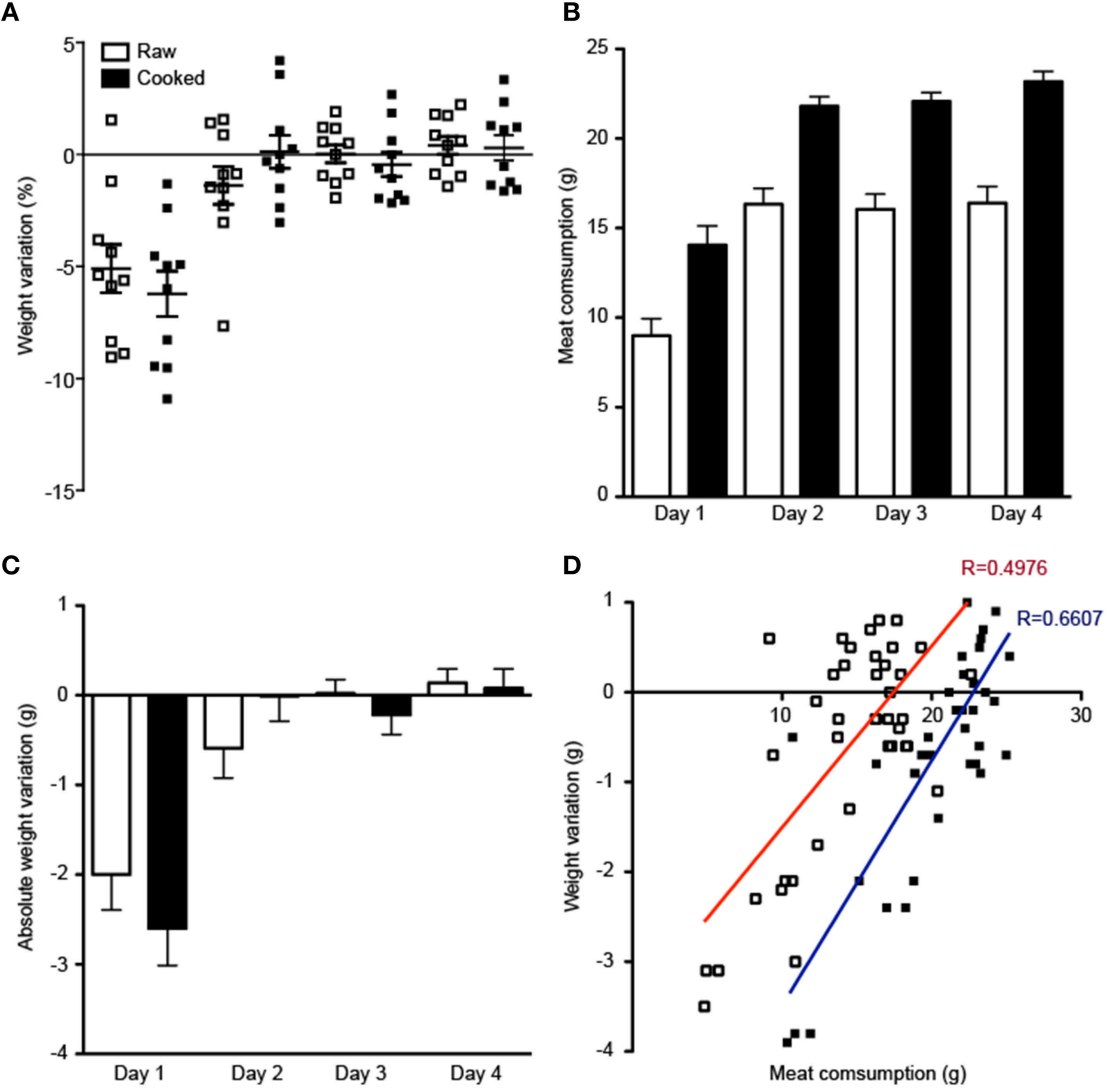
**Mice fed with cooked or raw meat present similar weight variations. (A)** Relative daily weight variation of mice fed exclusively on a raw meat diet (white squares) or cooked meat diet (black squares). **(B)** Average amount of meat consumed per day in groups fed with raw (white bar) or cooked (black bar) meat. **(C)** Absolute weight variation in both raw (white bar) and cooked (black bar) meat diet groups. Note that only on the first day there is a small decrease in the weight of both groups. **(D)** Correlation between weight variation and meat consumption during 4 days. Observe that the linear regression for the raw meat group (red) is shifted to the left, as compared to the cooked group (blue), indicating that for a similar variation of weight, animals fed on a raw meat diet require a lower amount of meat.

Unlike the lack of evidence of fire control, there is considerable evidence of the consumption of seeds (Richards, 2002; Strait et al., 2009) and meat (Heinzelin, 1999) dating back over 2 MY. Nevertheless, if we were to assume that thermal processing of food could arise at a similar stage and contribute to foraging efficiency, cooked meat should provide a higher caloric content than raw meat. To directly assess this possibility, we fed adult mice for 4 days with similar amounts of raw and cooked meat. As readout of the diet energetic content, we measured the animals weight every day and calculated whether the absolute or relative variation of weight correlated with meat consumption (Figure 4).

We observed a similar weight variation among animals fed with either cooked or raw meat (Figure 4A). Interestingly, animals fed with cooked meat ingested, on the average, more meat than animals fed with raw meat (Figure 4B). In both groups, ingestion of meat was lower on the first day, likely due to the adaptation to a new diet. As a consequence, animals from both groups lost around 2 g on this first day of the diet, but afterward their weight stabilized (Figure 4C). Finally, we correlated the weight variation and meat consumption for each animal (Figure 4D) and observed that, for a given value of weight variation, animals fed with cooked meat required a higher ingestion of food than animals fed with raw meat, probably reflecting the loss of fat caused by thermal processing (Sheard et al., 1998). Thus, our data indicate that energetic gain in a diet based exclusively on raw meat is similar to, or even higher than, a diet of cooked meat.

## Discussion

The disproportional increase in brain size relative to body size is a distinctive evolutionary feature of humans. Given the high metabolic cost of the human brain, many authors have suggested that dietary modifications played important roles during human evolution (Heinzelin, 1999; Richards, 2002; Strait et al., 2009; Organ et al., 2011; Wrangham, 2013). This conceivable metabolic constraint has been further extrapolated to suggest a tradeoff between brain and body size (Fonseca-Azevedo and Herculano-Houzel, 2012). Here, we show that energy intake is likely to have influenced human evolution, but not imposing a direct tradeoff between those variables. Furthermore, we provide direct evidence for the unlikelihood of thermal food processing as an important factor to increase calorie availability to sustain the increased number of brain neurons in the hominin lineage.

Energy intake is a direct product of the number of foraging hours (*H*) and average caloric content of food (i.e., foraging efficiency, *Q*). Here, we show that early hominins are likely to have obtained enough energy to sustain a large brain on a raw-food diet with 5–6 h of foraging per day. These observations are in clear contradiction with previous data in the literature suggesting that early hominins would require more than 9 h of foraging per day to sustain their body mass and number of neurons (Fonseca-Azevedo and Herculano-Houzel, 2012). We believe that such discrepancy can be explained by the fact that previous work underestimated the energy intake of hominins and, therefore, assumed an extremely limited foraging efficiency (~200–250 kcal/h) for early hominins (Fonseca-Azevedo and Herculano-Houzel, 2012). In fact, Fonseca-Azevedo and Herculano-Houzel (2012) used a *Z* factor of 70 to calculate the energetic needs of non-human primates, which likely underestimates the foraging efficiency of these species (Alperson-Afil et al., 2009). Furthermore, they took into account only the diet of non-human primates predominantly frugivorous or herbivores, which leads us to the conclusion that the equation used in their work to estimate foraging efficiency (*E*_*IN*_ = 25.352 × ${\text{M}}_{\text{BD}}^{0.526}$ × *H*) does not reflect the omnivorous diet of species in the hominin lineage (Richards, 2002).

Accordingly, pieces of evidence indicate that, compared to non-human primates, modern humans are clear evolutionary outliers for the number of hours spent foraging (Organ et al., 2011). For instance, hunter-gatherers, such as the Kalahari Bushmen and Hadza of Tanzania spend on the average 3–5 h per day foraging, obtaining from 2140 to 3000 kcal/day (Cohen, 2000). These values indicate a foraging efficiency of 430 (minimum) to 1000 kcal/h (maximum), which is far greater than that estimated by Fonseca-Azevedo and Herculano-Houzel (250 kcal/h) and closer to our estimates predicting that early hominins could easily afford their brain and body size spending about 5 h foraging. Foraging efficiency of early hominins could also have been deeply influenced by cooperative hunting and butchering (Dominguez-Rodrigo et al., 2010) as well as by new forms of communitarianism. These forms of cooperation, including egalitarianism, developing of sophisticated empathy and mind reading, language and cultural transmission are now considered key aspects of hominin evolution, as proposed by the “socio-cognitive niche” hypothesis (Whiten and Erdal, 2012). Thus, the enhancement of cognitive and behavioral complexity transformed our ancestors in the most efficient predators on Earth in spite of their lack of anatomical-hunting adaptations.

Early hominins likely increased their foraging efficiency by varying their diets, including seeds and meat, which are more caloric foods than wild plants and fruits. We provide compelling evidence indicating that thermal food processing is unlikely to explain increases in the foraging efficiency of early hominins. Firstly, there is no archeological evidence of fire control at the onset of brain expansion in the human lineage. Secondly, archeological data establishing a clear relationship between fire control and cooking by hominins are present only in the last 790,000 YA. Thirdly, thermal processing of meat does not increase energy content as indicated by variations in the body mass of mice fed in a raw or cooked meat diet. Although possible energetic benefits of cooked meat may have evolved slowly over time and not be appreciable in mice, our data in this animal model is important to refute previous data in the literature supporting the cooking hypothesis (Carmody et al., 2011). In this work, Carmody and colleagues suggest that the cooking of tubers and meat increases energetic gain in mice, as reflected by weight gain. Here, we repeated the same experiment with meat and failed to find such energetic benefit in the thermal processing of meat. Interestingly, Carmody and Wrangham (2009) have correctly pointed out in a previous work the paucity of studies directly comparing raw and cooked meat with respect to energy. They also highlight that results obtained from the few studies to date were often contradictory. Therefore, we conclude that more experiments need to be performed in order to address the possible energetic benefits of thermal processing of meat in humans.

Notably, even the guardians of theories stating that a relatively rapid increase in brain size, observed in the *Homo erectus*, is the result of cooking acknowledge the lack of archeological (Wrangham, 2013) evidence in their favor. Such theories must include mandatory evidence of control of fire associated with cooking, at least, 2 MYA, which is currently out of question.

Therefore, in this study, we favor the most classical view suggesting that early hominins increased their foraging efficiency by including new sources of food in their diet, especially seeds and meat (Milton, 1999; Richards, 2002). Also, the use of tools is well documented (Keeley and Toth, 1981; Domínguez-Rodrigo et al., 2005) and strongly correlates with the increase in brain size during human evolution (Klein, 1977; Holloway, 1997). Indeed, recent evidence indicates that the use of Lower Paleolithic technologies to process meat and tubers reduced the number of chewing cycles and the total masticatory force (Zink and Lieberman, 2016), explaining the increased foraging efficiency of early hominins at the onset of brain expansion. Therefore, the rise in the use of tools, rather than cooking, is more likely to explain how early hominins increased their daily energetic intake.

It is also possible that the emergence of new physiological mechanisms improved the capacity of early hominins to obtain energy from food intake. For instance, differential expression of glucose transporters in the human brain could facilitate energy allocation (Fedrigo et al., 2011) and even changes in the gut microbiota could contribute to raise the absorption of nutrients, thus increasing the caloric content obtained from food (Bäckhed et al., 2005).

In conclusion, the appealing hypothesis of thermal processing of food as a pre-requisite to brain expansion during evolution is not supported by archeological, physiological, and metabolic evidence. Most likely, the control of fire and cooking are rather a consequence of the emergence of a sophisticated cognition among hominins. We therefore, advocate that the hypothesis on what builds humankind uniqueness need consistent archeological and physiological data before being heralded.

## Author contributions

MC and AC wrote the manuscript. RDN and RDB revised archeological data. CQ, AC, and MC have worked in the mathematical model. AC collected data from animal experiments. MC designed the study and compiled results. All authors read and approved the final version of the manuscript.

### Conflict of interest statement

The authors declare that the research was conducted in the absence of any commercial or financial relationships that could be construed as a potential conflict of interest.
