# Supplementary material for: Human Brain Expansion during Evolution Is Independent of Fire Control and Cooking
Source: Front Neurosci. 2016 Apr 25;10:167. doi: 10.3389/fnins.2016.00167 (PMC4842772; doi:10.3389/fnins.2016.00167)
Supplement: Supplementary file 1 [file DataSheet1.docx]

Supplementary Material

**Human brain expansion during evolution is independent of fire control and cooking**

**Alianda Maira Cornélio^*^, Ruben Ernesto de Bittencourt-Navarrete, Ricardo de Bittencourt Brum, Claudio Marcos Queiroz and Marcos Romualdo Costa***

*** Correspondence:** Alianda M. Cornélio: alianda@neuro.ufrn.br

Marcos R. Costa: mrcosta@neuro.ufrn.br

# Supplementary Figures

**Supplementary Figure 1: Examples of raw and cooked diets used to feed adult mice.**

**Supplementary Figure 2: Expansion of brain volume among primates correlates with time but not with body mass.**  **A)** Maximal brain volume for different species of primates according to the mean time of existence in millions of years ago (MYA). The linear regression indicates that increase of brain volume correlates with time (R^2^=0.6277; p<0.0001). **B)** Maximal brain volume for different species of primates according to the body mass in kilograms (kg). The linear regression indicates that increase of brain volume does not correlate with body mass (R^2^=0.1888).
