# Supplementary figures and images for: Human Brain Expansion during Evolution Is Independent of Fire Control and Cooking
Source: Front Neurosci. 2016 Apr 25;10:167. doi: 10.3389/fnins.2016.00167 (PMC4842772; doi:10.3389/fnins.2016.00167)

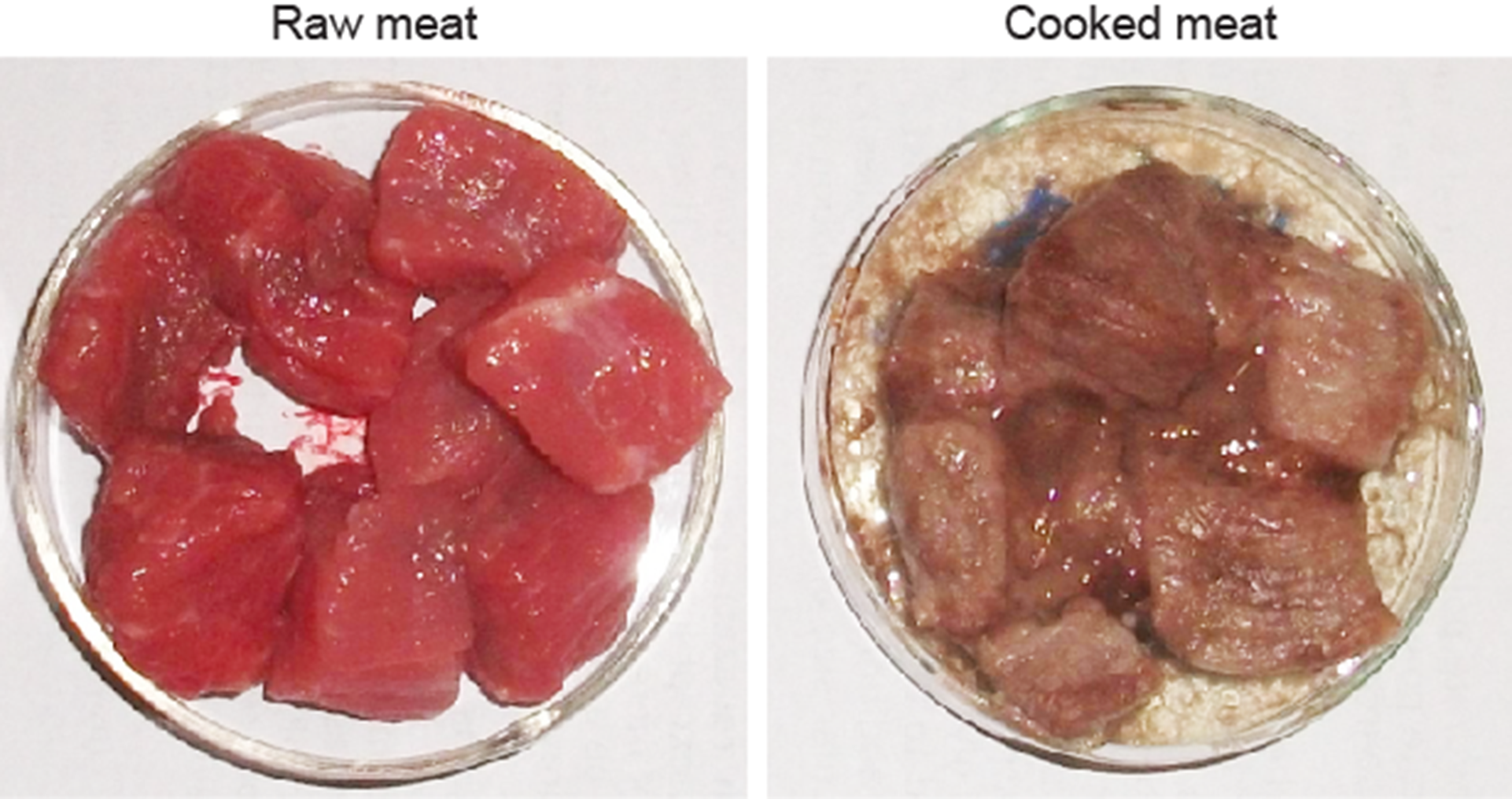

Supplement: Supplementary file 2 [file Image1.TIF]

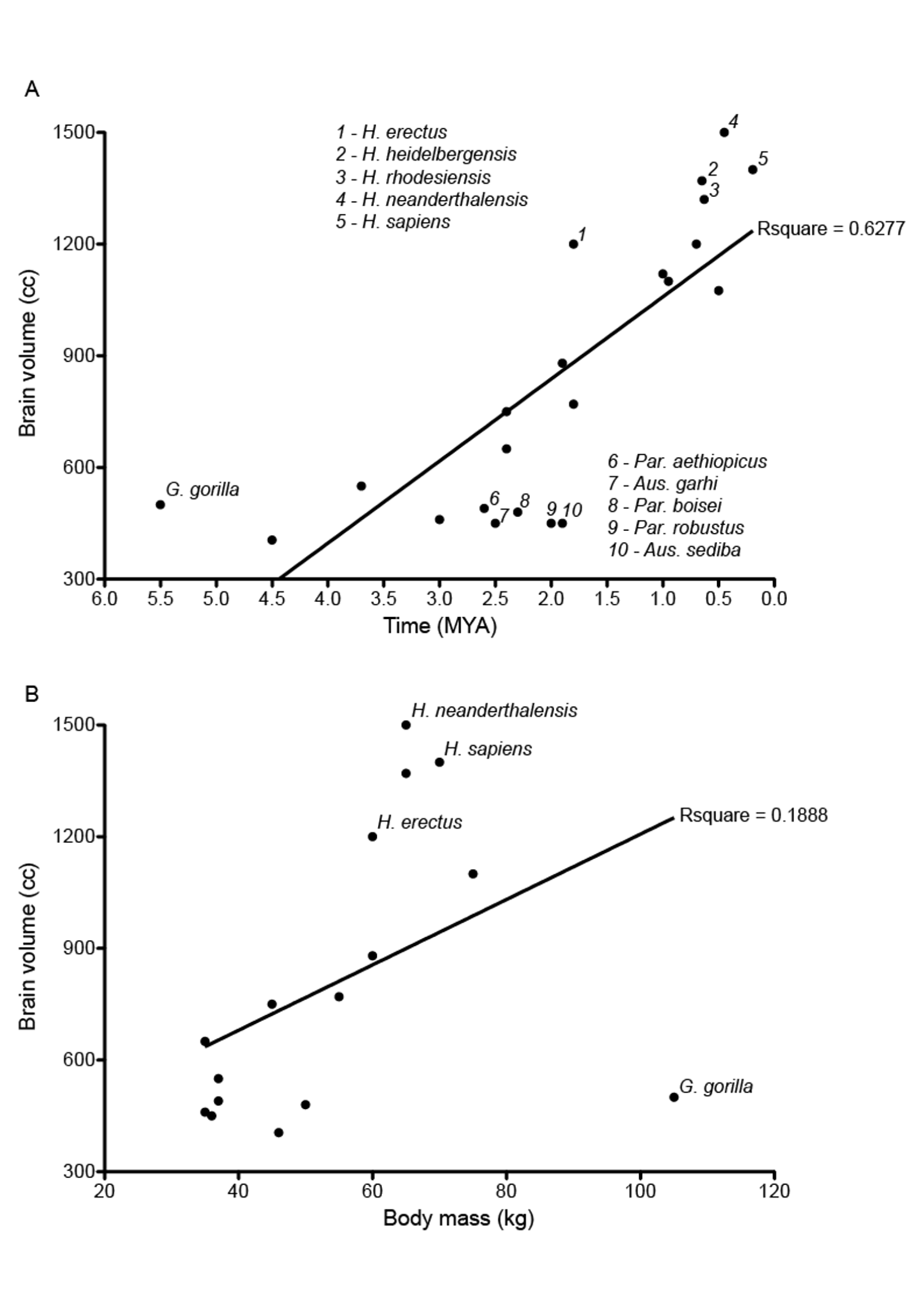

Supplement: Supplementary file 3 [file Image2.TIF]
